# Impact of antioxidant and micronutrient intake on varicocele-associated infertility: a retrospective analysis

**DOI:** 10.3389/fnut.2026.1838102

**Published:** 2026-06-15

**Authors:** Dian Fu, Xiaowen Tan, Zhigang Zheng, Xiaoming Yi, Changjie Shi, Ding Wu, Wei Zhang, Haowei He, Xuejun Shang

**Affiliations:** 1 Department of Urology, Nanjing Jinling Hospital, Affiliated Hospital of Medical School, Nanjing University, Nanjing, China; 2 Fuxing Road Outpatient Department, Jingnan Medical District of Chinese PLA General Hospital, Nanjing, China

**Keywords:** antioxidants, infertility, micronutrients, nutrition, varicocele

## Abstract

**Background:**

Varicocele is a leading cause of male infertility, primarily due to oxidative stress. This study aimed to investigate the relationship between dietary intake of antioxidants and micronutrients and semen quality in men with varicocele-related infertility.

**Methods:**

A retrospective analysis was conducted on 380 men between January 2018 and December 2023. Dietary intake of Vitamin C, E, Zinc, Selenium, CoQ10, and L-carnitine was assessed using a validated food frequency questionnaire. Semen parameters and DNA Fragmentation Index (DFI) were compared among intake tertiles (T1–T3) using ANOVA and multivariable linear regression, with adjustments for age, BMI, and smoking status.

**Results:**

A higher intake of antioxidants (T3) was found to be significantly associated with increased sperm concentration (38.79 vs. 33.70 million/ml, *p* < 0.001), total motility (51.09% vs. 46.00%, *p* < 0.001), and improved morphology (*p* < 0.001). Regression models indicated that Zinc and CoQ10 were strong predictors of concentration, while Vitamin E and L-carnitine significantly predicted progressive motility (*p* < 0.001). Interestingly, Vitamin C intake showed an inverse correlation with DNA fragmentation (β = −2.46, *p* < 0.001).

**Conclusion:**

The findings consistently show that higher intake of certain micronutrients is linked to better semen quality and lower DNA damage in men with varicocele. This underscores the importance of optimizing nutrition as a crucial approach in addressing infertility related to varicocele.

## 1 Introduction

Varicocele is a condition characterized by the abnormal enlargement of the pampiniform plexus due to the backflow of blood from the testicular vein, often caused by congenital defects in venous valves (1). It is a well-known reversible factor contributing to male infertility. Studies show that varicocele affects around 15% of men in the general population, but its prevalence is much higher in clinical settings. It is found in approximately 35–40% of men with primary infertility and up to 81% of those with secondary infertility (2, 3). The condition initiates a pathological sequence of events involving scrotal overheating, reduced oxygen levels, and venous blood flow backflow, which stimulates the excessive production of reactive oxygen species (ROS) (4, 5). When ROS levels surpass the body's natural defense mechanisms, the resulting oxidative stress disrupts sperm development, triggers cell death (apoptosis) (6), and more recently, sperm DNA damage. Chronic inflammation and decreased activity of antioxidant enzymes, such as superoxide dismutase (SOD), contribute to tissue damage and the buildup of hydrogen peroxide (H_2_O_2_) in varicocele patients. This oxidative stress is considered the main factor leading to decreased semen quality in these individuals (7–9).

The mainstay of conventional varicocele treatment is microsurgical varicocelectomy, which attempts to improve the testicular microenvironment and address venous reflux. Surgical intervention has been linked to increases in sperm concentration, but its effects on motility, morphology, and DNA integrity vary from person to person (10). Because of this diversity, there is a growing interest in complementary approaches that focus on the underlying causes, like oxidative stress.

In this regard, antioxidant therapy is becoming more widely acknowledged as a possible helpful strategy for controlling oxidative stress brought on by varicocele (11). L-carnitine and its derivative, acetyl-L-carnitine, are considered key therapeutic options due to their function as crucial mitochondrial “energy shuttles.” These compounds aid in the transportation of long-chain fatty acids into the mitochondrial matrix for β oxidation, thereby enhancing the bioenergetic capacity of sperm cells and directly improving total sperm count, concentration, and motility (12). However, despite the recognized physiological significance of L-carnitine, current evidence on its specific role in varicocele remains inconclusive, leading to a lack of universal recommendations. Further research is needed to determine the optimal dosage and patient selection criteria for this treatment approach (13). Micronutrients like Selenium and Zinc are important for enzymatic support. A deficiency in selenium can lead to higher levels of oxidative stress, as well as reduced testicular size, atrophy of the sperm ducts, and structural abnormalities during sperm maturation in the epididymis (14); while Zinc is associated with lower DNA fragmentation and supports sperm production (15). Zinc plays a crucial role in sperm motility and spermatogenesis through its functions as a cofactor, antiapoptotic agent, and antioxidant (16). Additionally, Coenzyme Q10 and Folic acid aid in energy production and DNA synthesis. Ascorbic acid, or vitamin C, is another powerful antioxidant with high antioxidant potential. A deficiency of ascorbic acid in semen can result in DNA fragmentation and a decline in other semen quality parameters (7). Its presence in seminal fluid plays a significant role in protecting spermatozoa from DNA peroxidation and enhancing sperm motility (17).

Although there is growing interest in antioxidant and micronutrient-based strategies, the majority of the evidence that is currently available comes from controlled supplementation trials, and there is relatively little information on regular food intake in clinical settings. Specifically, it is yet unknown how differences in the consumption of micronutrients and antioxidants in the diet may relate to the integrity of DNA and the quality of semen in men with varicocele. This is a significant gap since pharmaceutical interventions and dietary exposures may differ in terms of consistency and amount. Thus, the purpose of this retrospective analysis is to investigate the relationship between semen quality measures, such as DNA fragmentation, and dietary intake of specific antioxidants and micronutrients in men with varicocele-associated infertility.

## 2 Methodology

### 2.1 Study design and study population

This retrospective study explored the association between dietary antioxidant and micronutrient intake and semen quality indicators among men diagnosed with varicocele-related infertility. Medical data of patients visiting a tertiary andrology and reproductive medicine clinic between January 2018 and December 2023 were examined. Clinical, laboratory, and dietary data were retrospectively retrieved from records and study questionnaires obtained during the study period. The final analysis comprised 380 men with confirmed infertility and clinically diagnosed varicocele. Infertility was regarded as the failure to conceive after at least 12 months of consistent, unprotected sexual activity. A standardized physical examination was used to diagnose varicocele, and then scrotal Doppler ultrasonography was used to verify the diagnosis. The severity of varicocele was categorized using accepted clinical grading standards into Grades I, II, or III.

### 2.2 Eligibility criteria

Men between the ages of 20 and 45 who had primary or secondary infertility and a clinically confirmed varicocele were selected. Patients with established chromosomal or genetic reasons of infertility, endocrine disorders impacting reproductive function, active urogenital infections, or chronic systemic conditions that may influence semen characteristics were excluded. To reduce any confounding effects, people who had utilized antioxidant supplements, hormone therapy, or fertility-related drugs within 3 months of the semen examination were also disqualified. Records that lacked important clinical, nutritional, or laboratory information were also eliminated. Due to the retrospective design, internal comparisons across dietary consumption tertiles were used for analysis instead of a separate control or external comparison group.

### 2.3 Clinical and lifestyle variables

Demographic and clinical data was retrieved from electronic medical records, such as age, body mass index (BMI), duration of infertility, infertility type (primary or secondary), smoking status, and varicocele grade. The smoking status was classified as either non-smoker or current smoker. These factors were added to adjusted statistical models because they were thought to be possible confounders due to their established impact on male reproductive function.

### 2.4 Assessment of antioxidant and micronutrient intake

A validated semi-quantitative food frequency questionnaire was used to measure dietary intake of antioxidants and micronutrients linked to oxidative balance and male reproductive health during regular infertility screening. The questionnaire was modified from a previously validated semi-quantitative food frequency questionnaire created for adult populations, and it included 90 food items that represented significant dietary sources of antioxidants and micronutrients (18, 19, 30). Key nutrient consumption, such as vitamin C, vitamin E, zinc, selenium, coenzyme Q10, and L-carnitine, was recorded in the questionnaire. Standardized food composition tables were used to translate reported dietary consumption frequencies into predicted daily nutrient intake. The possibility of recall bias and inaccurate reporting cannot be ruled out because dietary intake was self-reported using a food frequency questionnaire.

The Institute of Medicine's (IOM) Dietary Reference Intakes (DRIs) for adult men used as the basis for the evaluation of intake levels. The following adequacy thresholds were used: zinc (11 mg/day), selenium (55 μg/day), vitamin C (90 mg/day), vitamin E (15 mg/day α-tocopherol), coenzyme Q10 (adequacy operationalized based on commonly reported dietary intake ranges ≥100 mg/day), and L-carnitine (adequacy defined using literature-based intake estimates ≥500 mg/day). Accordingly, participants were categorized as having a sufficient or inadequate intake. The total consumption of antioxidants was then divided into three tertiles.

#### 2.4.1 Definition of key terms

##### 2.4.1.1 Tertiles (T1–T3)

Tertile grouping was determined by dietary assessment of the combined intake of vitamin C, vitamin E, zinc, selenium, CoQ10, and L-carnitine.

##### 2.4.1.2 Nutrients contributing to tertiles

Adequate intake is defined as nutritional consumption that meets or exceeds specified dietary reference values recommended dietary intake/acceptable intake (RDI/AI). Values below these limits were regarded as insufficient.

##### 2.4.1.3 Adequate intake

Adequate intake is defined as nutrient consumption levels that meet or surpass established dietary recommendations based on accepted nutritional guidelines. In this study, adequacy was established by comparing predicted daily intake values to recommended dietary intake (RDI) and acceptable intake (AI) levels. Participants were classed as “adequate” or “inadequate” in terms of their consumption.

##### 2.4.1.4 Antioxidant intake

Antioxidant intake refers to the total dietary consumption of substances that help reduce oxidative stress, such as vitamin C, vitamin E, CoQ10, and L-carnitine.

##### 2.4.1.5 Micronutrient intake

Micronutrient intake refers to the consumption of key vitamins and trace elements needed for optimal physiological and reproductive function, specifically those investigated in this study.

### 2.5 Semen analysis and DNA fragmentation assessment

Semen samples were obtained after 2–5 days of sexual abstinence and examined in a certified andrology laboratory using standard operating procedures suggested by the World Health Organization laboratory manual for semen evaluation. The metrics that were assessed were sperm concentration (million/mL), total motility (%), progressive motility (%), and normal morphology (%). The terminal deoxynucleotidyl transferase-mediated dUTP nick-end labeling (TUNEL) test was used to analyze sperm DNA fragmentation, an extensively recognized approach for detecting DNA strand breaks in spermatozoa. The data were presented as the DNA Fragmentation Index (DFI), which is the percentage of sperm with fragmented DNA. Abnormal DNA fragmentation was defined as ≥25% DFI using clinical reference values.

### 2.6 Statistical analysis

Categorical parameters were displayed as frequencies and percentages, whilst continuous factors were displayed as mean ± standard deviation. One-way analysis of variance (ANOVA) was used to assess variations in semen parameters among antioxidant consumption tertiles, Tukey's *post-hoc* test was then used for pairwise comparisons. After that, multivariable linear regression models were built to evaluate the relationships between specific micronutrients and outcomes related to semen quality while controlling for potential confounders like age, BMI, and smoking status. Regression coefficients (β), 95% confidence intervals, and accompanying *p*-values were computed to determine effect sizes and statistical significance. IBM SPSS Statistics version 26.0 (IBM Corp., Armonk, NY, USA) was used for all statistical tests; a two-tailed *p*-value of less than 0.05 was deemed statistically significant.

## 3 Results

### 3.1 Participant characteristics

A total of 380 men with varicocele-related infertility were included in the retrospective study. The mean age of the individuals was 32.77 ± 4.91 years, and their body mass index (BMI) was 24.82 ± 2.90 kg/m^2^. The average length of infertility was almost 4 years. The majority of cases were primary infertility, whereas a lesser percentage of the cohort had secondary infertility. Roughly one-third of the individuals stated that they currently smoke. The most common type of varicocele in terms of severity was Grade II, which was followed by Grade I and Grade III (Table 1).

**Table 1:** Baseline characteristics of study participants.

Characteristic	Total
Sample size	380
Age (years)	32.77 ± 4.91
BMI (kg/m^2^)	24.82 ± 2.90
Infertility duration (years)	4.05 ± 1.38
Primary infertility	274 (72.1%)
Secondary infertility	106 (27.9%)
Smokers	121 (31.8%)
Varicocele grade I	110 (28.9%)
Varicocele grade II	187 (49.2%)
Varicocele grade III	83 (21.8%)

### 3.2 Patterns of antioxidant and micronutrient intake

Antioxidant and micronutrient intake assessments showed a moderate degree of variation among the participants. Zinc and vitamin C intake were most frequently found to be adequate, while a sizable percentage of subjects also had enough selenium intake. About 50% of the people consumed enough vitamin E and coenzyme Q10 to meet recommended levels. Adequate L-carnitine intake, on the other hand, was comparatively less common in the sample (Table 2).

**Table 2:** Prevalence of adequate antioxidant intake.

Antioxidant	Adequate intake *n* (%)
Vitamin C	221 (58.16%)
Vitamin E	192 (50.53%)
Zinc	235 (61.84%)
Selenium	212 (55.79%)
Coenzyme Q10	195 (51.32%)
L-Carnitine	175 (46.05%)

### 3.3 Relationship between antioxidant intake and semen quality indicators

**Figure 1:**
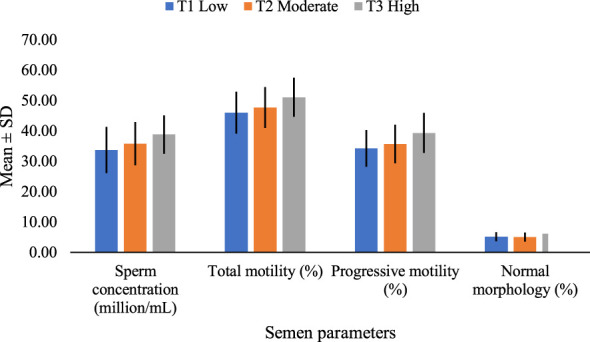
Semen parameters according to antioxidant intake.

A progressive trend of improvement in a number of semen measures was found when examining semen properties across increasing amounts of antioxidant intake. Sperm concentration tended to be higher in men who consumed more antioxidants than in those who consumed less. Both total and progressive sperm motility showed a similar pattern, with those in the higher intake group exhibiting better motility profiles. Individuals who consumed more antioxidants also exhibited a little tendency toward improved normal sperm morphology (Tables 3, 4 and Figure 1).

**Table 3:** Semen parameters according to antioxidant intake.

Parameter	T1 low (*n* = 119)	T2 moderate (*n* = 128)	T3 high (*n* = 133)	*p*-value
Sperm concentration (million/ml)	33.70 ± 7.61	35.81 ± 7.11	38.79 ± 6.33	<0.001
Total motility (%)	46.00 ± 6.91	47.71 ± 6.74	51.09 ± 6.42	<0.001
Progressive motility (%)	34.23 ± 6.03	35.67 ± 6.35	39.34 ± 6.57	<0.001
Normal morphology (%)	5.14 ± 1.47	5.03 ± 1.50	6.13 ± 1.37	<0.001

**Table 4:** *Post hoc* test.

Parameter	T1 vs. T2	T1 vs. T3	T2 vs. T3
Sperm concentration	0.054	<0.001	0.002
Total motility	0.107	<0.001	<0.001
Progressive motility	0.173	<0.001	<0.001
Normal morphology	0.822	<0.001	<0.001

### 3.4 Associations between individual micronutrients and semen parameters

Multivariable regression analyses were performed while controlling for relevant confounders, such as age, BMI, and smoking status, in order to further investigate potential associations. Sperm concentration seems to be positively correlated with zinc intake, while coenzyme Q10 intake showed a similar relationship. Consumption of selenium was linked to overall sperm motility. Furthermore, progressive sperm motility, a measure strongly related to sperm functional capacity, seemed to be related with vitamin E and L-carnitine intake. On the other hand, vitamin C intake showed an inverse relationship with sperm DNA fragmentation (Table 5).

**Table 5:** Multivariable linear regression for semen parameters.

Micronutrient	Outcome	β coefficient	95% *CI*	*p*-value
Zinc	Sperm concentration	1.77	0.25–3.29	0.023
Selenium	Total motility	1.84	0.42–3.25	0.011
Vitamin E	Progressive motility	3.97	2.68–5.27	<0.001
Vitamin C	DNA fragmentation	−2.46	−3.61–−1.32	<0.001
CoQ10	Sperm concentration	2.51	1.05–3.97	<0.001
L-Carnitine	Progressive motility	3.53	2.21–4.84	<0.001

### 3.5 Antioxidant intake and sperm DNA integrity

**Figure 2:**
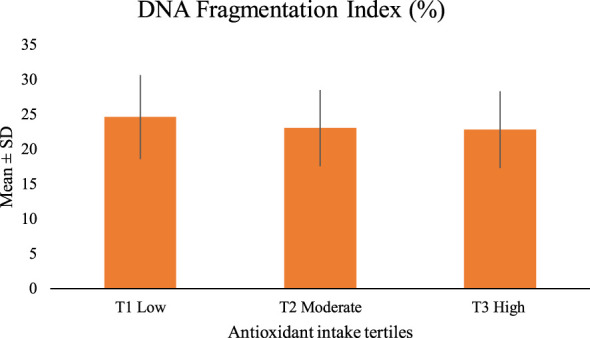
DNA fragmentation by antioxidant intake tertile.

Sperm DNA fragmentation generally decreased as antioxidant consumption increased. DNA fragmentation was usually lower in individuals who consumed more antioxidants than in those who did not, and the pattern across classes was statistically significant. Even though these outcomes should be treated cautiously owing to the retrospective design of the study, the pattern found indicates that higher antioxidant consumption may be correlated to lower oxidative stress-induced DNA damage in spermatozoa among men with varicocele-related infertility (Table 6 and Figure 2).

**Table 6:** DNA fragmentation by antioxidant intake tertile.

Tertile	DNA fragmentation index (%)	*p*-trend
T1 (low)	24.63 ± 6.03	
T2 (moderate)	23.04 ± 5.47	
T3 (high)	22.82 ± 5.52	<0.05

## 4 Discussion

This retrospective study reveals that higher dietary antioxidant and micronutrient intake may be associated with better sperm quality measures in men with varicocele-induced infertility. Participants at the highest intake tertile appeared to have higher sperm quantity, motility, morphology, and less DNA fragmentation. Given that varicocele is characterized by increased oxidative stress, venous stasis, and mitochondrial dysfunction, these findings may indicate a link between dietary exposure and the testicular microenvironment, which influences spermatogenesis.

The progressive trend observed across antioxidant intake tertiles could indicate that increased dietary exposure to redox-active foods is linked to enhanced sperm characteristics. Excess reactive oxygen species in varicocele can reduce sperm production, compromise membrane integrity, and damage DNA. As a result, it is probable that persons who ingest more antioxidants will have a more balanced oxidative state. These findings are largely comparable with those reported by Nguyen et al. (20), who showed increased sperm concentration and decreased DNA fragmentation after vitamin supplementation. Similarly, Busetto et al. (21) found that antioxidant-based therapies enhanced sperm parameters, notably in mild to severe varicocele cases. While those studies were interventional, the current findings may represent similar trends within habitual dietary intake, albeit causality cannot be determined.

Individual micronutrients, CoQ10 and zinc, were found to be linked with sperm concentration. CoQ10 is essential for mitochondrial electron transport and cellular energy production, and its reduced form (ubiquinol) also serves as an antioxidant capable of neutralizing reactive oxygen species (22, 23). Salvio et al. (24) demonstrate that CoQ10 supplementation may improve sperm parameters via mitochondrial and antioxidant mechanisms. Furthermore, Bakri et al.'s (25) meta-analysis discovered that infertile men's sperm concentrations improved after consuming CoQ10. In the current investigation, dietary CoQ10 intake may also be associated with sperm density, particularly in the context of varicocele-related oxidative stress, however this connection is just associative. Zinc also correlated positively with sperm concentration, which is consistent with its role in DNA synthesis, chromatin integrity, and antioxidant protection. Nguyen et al. (20) also showed improvements in zinc status and semen quality, implying that micronutrient adequacy may be important in populations with inadequate intake.

Vitamin E and L-carnitine correlations with increasing motility may provide more evidence for the role of oxidative and metabolic pathways in sperm activity. Vitamin E, a lipid-soluble antioxidant, is hypothesized to protect sperm membrane integrity from lipid peroxidation, whereas L-carnitine promotes mitochondrial fatty acid transport and energy metabolism. These pathways are especially important in varicocele, where mitochondrial failure and oxidative damage are common. Ener et al. (26) discovered that vitamin E supplementation after varicocelectomy improved semen parameters, while Saeedian et al. (27) found increased motility with vitamin E intervention. Similarly, Li et al. (28) identified L-carnitine as one of the more effective therapies for enhancing sperm motility using a network meta-analysis, while Tsampoukas et al. (13) proposed L-carnitine's possible function in infertile males with varicocele. The current findings may reflect comparable trends at the nutritional level, although they should be viewed with caution.

An inverse relationship between vitamin C intake and sperm DNA fragmentation was also discovered, implying a possible link between increased antioxidant intake and lower oxidative DNA damage. Sperm DNA integrity is especially sensitive to oxidative stress due to a lack of innate antioxidant defenses. Gual-Frau et al. (7) discovered that antioxidant therapy improved sperm DNA integrity in varicocele patients, while Saber-Khalaf et al. (29) found that antioxidant treatment reduced DNA fragmentation, however surgical intervention had a greater impact. In this context, the current findings suggest that dietary vitamin C intake is associated with lower DNA fragmentation, although this association should be viewed as associative rather than causal.

### 4.1 Strengths

- One major strength of this study is the very large cohort of males with clinically diagnosed varicocele-related infertility, which allows for more reliable estimations when compared to smaller studies.
- The combination of standard semen characteristics with DNA fragmentation allows a more complete assessment of sperm quality.
- Furthermore, tertile-based comparisons allow for the detection of potential dose-response connections, while multivariable regression analysis takes into account major confounders such as age, BMI, and smoking status.

### 4.2 Limitations

- Due to the retrospective design of our study, we can establish strong correlations but cannot conclusively prove that these nutrients directly caused the changes in semen parameters.
- Our study was based on self-reported dietary data, which may be influenced by recall bias in terms of accurately remembering nutrient intake.
- As a single-center study, our results are limited to a specific population and may not be broadly applicable to global dietary patterns or diverse environments.
- Additional potential confounders, such as alcohol consumption and a history of varicocelectomy, were not considered in the analysis, which could have resulted in residual confounding.
- Furthermore, the study did not examine fertility outcomes such as clinical pregnancy or live birth.
- The lack of a control or comparison group limits the capacity to identify the precise contribution of dietary consumption, hence the results should be taken as associative rather than causal.

Overall, our findings imply that males with varicocele-associated infertility benefit from a higher dietary intake of particular antioxidants and minerals, as well as reduced DNA fragmentation. While these findings suggest a link between nutritional variables and reproductive health, more prospective and interventional research are needed to better understand these associations and their clinical consequences.

## 5 Conclusion

In conclusion, our retrospective analysis of 380 patients has identified specific dietary micronutrients, such as L-carnitine, Coenzyme Q10, and Vitamin C, as significant independent predictors of sperm quality in men with varicocele. The study highlights the importance of addressing nutritional deficiencies, particularly in L-carnitine and Zinc, to improve reproductive outcomes by reducing oxidative stress and genomic damage. These findings suggest that targeted dietary optimization is a cost-effective and high-yield strategy for enhancing fertility in men with varicocele.

## Data Availability

The raw data supporting the conclusions of this article will be made available by the authors, without undue reservation.

## References

[1] Shi S Chen W Tian J Liang Z Wu J Li J . Risk factors associated with varicocele: a narrative review. Transl Androl Urol. (2025) 14:1807–17. doi: 10.21037/tau-2025-120 40687657 PMC12271938

[2] Sasson DC Kashanian JA. Varicoceles. JAMA. (2020) 323:2210. doi: 10.1001/jama.2020.0397 32484535

[3] Omar MI Pal RP Kelly BD Bruins HM Yuan Y Diemer T . Benefits of empiric nutritional and medical therapy for semen parameters and pregnancy and live birth rates in couples with idiopathic infertility: a systematic review and meta-analysis. Eur Urol. (2019) 75:615–25. doi: 10.1016/j.eururo.2018.12.022 30630643

[4] Ghaleno LR Alizadeh A Drevet JR Shahverdi A Valojerdi MR. Oxidation of sperm DNA and male infertility. Antioxidants. (2021) 10:97. doi: 10.3390/antiox10010097 33445539 PMC7827380

[5] Jensen CFS Østergren P Dupree JM Ohl DA Sønksen J Fode M. Varicocele and male infertility. Nat Rev Urol. (2017) 14:523–33. doi: 10.1038/nrurol.2017.98 28675168

[6] Ritchie C Ko EY. Oxidative stress in the pathophysiology of male infertility. Andrologia. (2020) 53:e13581. doi: 10.1111/and.13581 32323352

[7] Gual-Frau J Abad C Amengual MJ Hannaoui N Checa MA Ribas-Maynou J . Oral antioxidant treatment partly improves integrity of human sperm DNA in infertile grade I varicocele patients. Hum Fertil. (2015) 18:225–9. doi: 10.3109/14647273.2015.1050462 26090928

[8] Hassani-Bafrani H Najaran H Razi M Rashtbari H. Berberine ameliorates experimental varicocele-induced damages at testis and sperm levels; evidences for oxidative stress and inflammation. Andrologia. (2018) 51:e13179. doi: 10.1111/and.13179 30334274

[9] Babaei A Asadpour R Mansouri K Sabrivand A Kazemi-Darabadi S. Lycopene protects sperm from oxidative stress in the experimental varicocele model. Food Sci Nutr. (2021) 9:6806–17. doi: 10.1002/fsn3.2632 34925809 PMC8645712

[10] Takács T Szabó A Kopa Z. Recent trends in the management of varicocele. J Clin Med. (2025) 14:5445. doi: 10.3390/jcm14155445 40807066 PMC12347406

[11] Szymański M Domaracki P Szymańska A Wandtke T Szyca R Brycht Ł . The role and place of antioxidants in the treatment of male infertility caused by varicocele. J Clin Med. (2022) 11:6391. doi: 10.3390/jcm11216391 36362619 PMC9655278

[12] Kumar R Garg H. An update on the role of medical treatment including antioxidant therapy in varicocele. Asian J Androl. (2016) 18:222. doi: 10.4103/1008-682X.171657 26763549 PMC4770490

[13] Tsampoukas G Khan MF Katsouri A Akhter W Moussa M Deliveliotis K . L-carnitine as primary or adjuvant treatment in infertile patients with varicocele. A systematic review. Archivio Italiano Di Urologia E Andrologia. (2020) 92:523–3. doi: 10.4081/aiua.2020.3.263 33016059

[14] Walczak–Jedrzejowska R Wolski JK Slowikowska–Hilczer J. The role of oxidative stress and antioxidants in male fertility. Cent Eur J Urol. (2013) 65:60–7. doi: 10.5173/ceju.2013.01.art19 PMC3921845 24578993

[15] Cui X Jing X Wu X Yan M Li Q Shen Y . DNA methylation in spermatogenesis and male infertility. Exp Ther Med. (2016) 12:1973–9. doi: 10.3892/etm.2016.3569 27698683 PMC5038464

[16] Nematollahi-Mahani SN Azizollahi GH Baneshi MR Safari Z Azizollahi S. Effect of folic acid and zinc sulphate on endocrine parameters and seminal antioxidant level after varicocelectomy. Andrologia. (2013) 46:240–5. doi: 10.1111/and.12067 23356505

[17] Asadi N. The impact of oxidative stress on testicular function and the role of antioxidants in improving it: a review. J Clin Diagn Res. (2017) 11:IE01–5. doi: 10.7860/JCDR/2017/23927.9886 28658802 PMC5483704

[18] Willett WC Sampson L Stampfer MJ Rosner B Bain C Witschi J . Reproducibility and validity of a semiquantitative food frequency questionnaire. Am J Epidemiol. (1985) 122:51–65. doi: 10.1093/oxfordjournals.aje.a114086 4014201

[19] Hu FB Rimm E Smith-Warner SA Feskanich D Stampfer MJ Ascherio A . Reproducibility and validity of dietary patterns assessed with a food-frequency questionnaire. Am J Clin Nutr. (1999) 69:243–9. doi: 10.1093/ajcn/69.2.243 9989687

[20] Nguyen ND Le MT Tran NQT Nguyen QHV Cao TN. Micronutrient supplements as antioxidants in improving sperm quality and reducing DNA fragmentation. Basic Clin Androl. (2023) 33:23. doi: 10.1186/s12610-023-00197-9 37704942 PMC10500740

[21] Busetto GM Agarwal A Virmani A Antonini G Ragonesi G Del Giudice F . Effect of metabolic and antioxidant supplementation on sperm parameters in oligo-astheno-teratozoospermia, with and without varicocele: a double-blind placebo-controlled study. Andrologia. (2018) 50:e12927. doi: 10.1111/and.12927 29315686

[22] Gutierrez-Mariscal FM Larriva APA Limia-Perez L Romero-Cabrera JL Yubero-Serrano EM López-Miranda J. Coenzyme Q10 supplementation for the reduction of oxidative stress: clinical implications in the treatment of chronic diseases. Int J Mol Sci. (2020) 21:7870. doi: 10.3390/ijms21217870 33114148 PMC7660335

[23] Alahmar AT Singh R. Comparison of the effects of coenzyme Q10 and Centrum multivitamins on semen parameters, oxidative stress markers, and sperm DNA fragmentation in infertile men with idiopathic oligoasthenospermia. Daehan Saengsik Uihak Hoeji/Clin Exp Reprod Med. (2022) 49:49–56. doi: 10.5653/cerm.2021.04910 PMC8923633 35255658

[24] Salvio G Cutini M Ciarloni A Giovannini L Perrone M Balercia G. Coenzyme Q10 and male infertility: a systematic review. Antioxidants. (2021) 10:874. doi: 10.3390/antiox10060874 34070761 PMC8226917

[25] Bakri S Saleh R Cayan S Birowo P Atmoko W Zainal ATF . Efficacy and safety of coenzyme Q10 in idiopathic male infertility: a systematic review and meta-analysis of randomized trials. World J Men Health. (2025) 43:1807–17. doi: 10.5534/wjmh.250159 PMC13646325 40878114

[26] Ener K Aldemir M Işik E Okulu E Özcan MF Ugurlu M . The impact of vitamin E supplementation on semen parameters and pregnancy rates after varicocelectomy: a randomised controlled study. Andrologia. (2016) 48:829–34. doi: 10.1111/and.12521 26780969

[27] Saeedian K Davaryar S Emadzadeh M Rezayat AA. The impact of vitamin E supplementation on sperm analysis in varicocelectomy patients: a triple-blind randomized controlled trial. Trials. (2025) 26:36. doi: 10.1186/s13063-025-08740-x 39891146 PMC11786367

[28] Li K Yang X Wu T. The effect of antioxidants on sperm quality parameters and pregnancy rates for idiopathic male infertility: a network meta-analysis of randomized controlled trials. Front Endocrinol. (2022) 13:810242. doi: 10.3389/fendo.2022.810242 PMC8898892 35265037

[29] Saber-Khalaf M Mohamed O Mahmoud O Abdelrazek M Taha EA Hosny A . Varicocelectomy versus antioxidants in infertile men with isolated teratozoospermia: a randomized controlled trial. Daehan Saengsik Uihak Hoeji/Clin Exp Reprod Med. (2025) 53:47–53. doi: 10.5653/cerm.2024.07493 PMC12954053 40659542

[30] Minhas S Boeri L Capogrosso P Cocci A Corona G Dinkelman-Smit M . European association of urology guidelines on male sexual and reproductive health: 2025 update on male infertility. Eur Urol. (2025) 87:601–16. doi: 10.1016/j.eururo.2025.02.026 40118737

